# A multivalent nanobody–drug conjugate to prevent and treat influenza virus infections

**DOI:** 10.1073/pnas.2409565122

**Published:** 2025-11-03

**Authors:** Thibault J. Harmand, Laura Pietrok, Helen Rich, Rhogerry Deshycka, Laney Flanagan, Aaron Accardo, Novalia Pishesha, Hidde L. Ploegh

**Affiliations:** ^a^Cerberus Therapeutics, Cambridge, MA 02139; ^b^Division of Immunology, Boston Children’s Hospital, Boston, MA 02115; ^c^Department of Pediatrics, Harvard Medical School, Boston, MA 02115; ^d^Program in Cellular and Molecular Medicine, Boston Children’s Hospital, Boston, MA 02115

**Keywords:** bioconjugate, nanobody drug conjugates, influenza

## Abstract

We describe the production and use of nanobody drug conjugates that comprise of VHH_kappa_, a nanobody that recognizes mouse immunoglobulin kappa light chains, and one or more copies of the small molecule influenza virus neuraminidase (NA) inhibitor, zanamivir. Such compounds achieve half-life extension of zanamivir, while recruiting polyclonal immunoglobulins of all isotypes regardless of specificity to infected cells for antibody-dependent cell-mediated cytotoxicity and complement-dependent cellular cytotoxicity. Since the influenza A virus (IAV) NA is a tetramer, we produced VHH_kappa_ adducts with 1, 2, or 4 zanamivir molecules attached in a site-specific manner, to allow multivalent engagement of NA. Administration of a VHH_kappa_ adduct modified with 4 zanamivir molecules (VHH_kappa_–Zan_4_) was ~10-fold more potent in protection against infection with IAV than VHH_kappa_–Zan carrying only a single zanamivir molecule. VHH_kappa_–Zan_4_ can be given intranasally to confer full protection against a lethal IAV challenge. The neutralizing antibody titers in the respiratory mucosa and in the circulation, as well as the serum IgG antibody response against the hemagglutinin and nucleoprotein, are higher in VHH_kappa_–Zan_4_ treated mice that survived the lethal challenge than in controls infected with a sublethal dose of virus. VHH_kappa_–Zan_4_ affords protection even when given intranasally weeks prior to a challenge with a lethal dose of IAV. This type of adduct can therefore be applied prophylactically and therapeutically and does not require prior immunization for protection against a lethal dose of IAV.

## Significance

An anti-influenza therapeutic was developed, based on the use of a nanobody that recognizes all mouse immunoglobulin kappa light chains (VHH_kappa_), covalently modified with 4 molecules of the influenza neuraminidase inhibitor zanamivir. This compound, VHH_kappa_–(zanamivir)_4_, can be given intranasally and protects influenza-infected mice when given 3 d after challenge with a lethal dose of the virus. The compound is effective even when given 2 wk prior to a challenge with a lethal dose of virus and protects against both A and B strains of the virus. This type of nanomedicine may find clinical application when translated to a human setting.

Immunotherapy may well be one of the most cost-effective forms of therapy and disease prevention. Vaccines, the earliest form of immunotherapy in clinical use, have pushed several viral pathogens to the brink of extinction or even eradicated them (1). What is required for a successful vaccine to elicit the desired response is delivery of the relevant antigen under inflammatory conditions, either through the deployment of attenuated strains of a pathogen or by inclusion of an adjuvant (2, 3). In preclinical models, intraperitoneal or intravenous delivery routes are commonly used, while clinical deployment relies on intramuscular or subcutaneous injection, oral delivery, or delivery by inhalation. Where possible, the latter two routes are preferred, because they avoid the use of needles. For a vaccine to generate a protective antiviral immune response may take weeks or more, complicating the treatment of immunocompromised or unvaccinated individuals already infected. On the other hand, small molecule antivirals used to combat influenza virus act rapidly, but their pharmacokinetics are not always favorable, as in the case of drugs such as the neuraminidase (NA) inhibitors oseltamivir and zanamivir (4).

Identifying clear correlates of protection to infections is challenging. The presence of microbicidal or neutralizing antibodies specific for the pathogen is often used as a proxy for protection following vaccination. The effector functions of immunoglobulins, carried in their Fc portion, allow the recruitment of Fc receptor-positive (FcR+) cells with cytotoxic and cytokine producing activity (antibody-dependent cell mediated cytotoxicity; ADCC), as well as the activation of complement for direct lytic activity or through recruitment and activation of cytotoxic cells (complement-dependent cell mediated cytotoxicity; CDC) (5–7).

The discovery that camelids make both conventional four-chain (HL)_2_-type immunoglobulins and heavy chain only (H)_2_ type immunoglobulins enabled the recombinant expression of just the variable region of these “heavy chain only” immunoglobulins to yield products referred to as Single variable domain on a heavy chain (VHHs) or nanobodies (8, 9). The discovery and application of nanobodies have been expertly reviewed (10–12). The appeal of nanobodies for both therapeutic and diagnostic applications lies in their small size, ease of expression, and modification, as well as in their stability. Their single-domain nature also implies that the mode of monovalent antigen recognition is distinct from that of typical immunoglobulins and may provide access to epitopes not as readily accessible to the heavy chain-light chain combination of conventional antibodies (13–16).

Nanobodies appear ideally suited to direct nanomedicines to their intended target. Their ease of site-specific modification, whether enabled by the installation of an unpaired cysteine or by enzymatic modification, has led to the design of nanoparticles that can be targeted to the site(s) recognized by the nanobodies attached to them (17). The conversion of nanobodies into theranostics through installation of the appropriate radioisotopes is a promising application (18, 19), because the short circulatory half-life of nanobodies limits systemic radiation exposure, while the specificity of nanobodies allows their significant enrichment at the sites targeted, such as infected organs or tumors. Along the same lines, modification of nanobodies with cytotoxic or immunosuppressive drugs (19–25) creates compounds with a short circulatory half-life and likewise limited systemic exposure, while still affording a therapeutic benefit through on-target efficacy.

In earlier work, we discovered a surprising application for nanobodies that recognize immunoglobulin light chains (Igκ) of mouse or human origin. We equipped antilight chain nanobodies (VHH_kappa_) with a small molecule influenza virus NA inhibitor, zanamivir (26). Influenza virus critically relies on the activity of its NA to ensure the release of newly formed virions (27). Because zanamivir is a strict sialic acid analog, the emergence of resistance to it is rare (28, 29). The NAs of both influenza A (IAV) and influenza B (IBV) strains are sensitive to zanamivir (30). The drug is administered within 48 h of diagnosis by inhalation, or it is given by intravenous injection to critically ill patients (30). As a small molecule, zanamivir is rapidly eliminated from the circulation and thus requires repeated dosing.

The conjugation of zanamivir to the C-terminus of VHH_kappa_ achieves half-life extension of the drug, as the adduct will bind to immunoglobulins of all isotypes in the circulation, regardless of their specificity. The circulatory half-life of VHH_kappa_–Zan in mice is 84.1 h (26). For comparison, the circulatory half-lives of immunoglobulins in mice are as follows: IgM ~ 2 d, IgG1 and IgG3 6 to 8 d, IgG2b 4 to 6 d, IgA < 4 d, and IgE 12 h. This covalent complex of VHH_kappa_ and zanamivir is required for activity, as a mixture of zanamivir and VHH_kappa_ does not afford protection from lethal IAV infection (26). Mere half-life extension of zanamivir also did not fully account for this protective effect, as a similar adduct comprising an antiserum albumin VHH and zanamivir showed a similar circulatory half-life, but required a far higher dose for protection (26). This suggests that extension of circulatory half-life renders zanamivir more effective than the drug alone, but it does not account for the level of protection seen with VHH_kappa_–Zan. The mechanism responsible for protection requires the presence of immunoglobulins. VHH_kappa_–Zan fails to protect RAG-deficient mice from a lethal IAV infection, which lack mature T and B cells, unless they also receive a dose of polyclonal Ig (26). VHH_kappa_–Zan also brings infected cells and/or viral particles into close contact with circulating immunoglobulins of all isotypes. Thanks to the diverse effector functions of immunoglobulin Fc portions, these immunoglobulins facilitate the recruitment of a broad array of FcR+ cells (such as natural killer cells, macrophages, neutrophils, dendritic cells, etc.) involved in ADCC and CDC.

In this work, we expand on this preliminary concept and design nanobody-based antivirals that can be given intranasally to achieve full protection, even when administered weeks prior to infection.

## Results and Discussion

### Structurally Defined Multizanamivir Molecules Installed on VHH_kappa_.

**Fig. 1.**
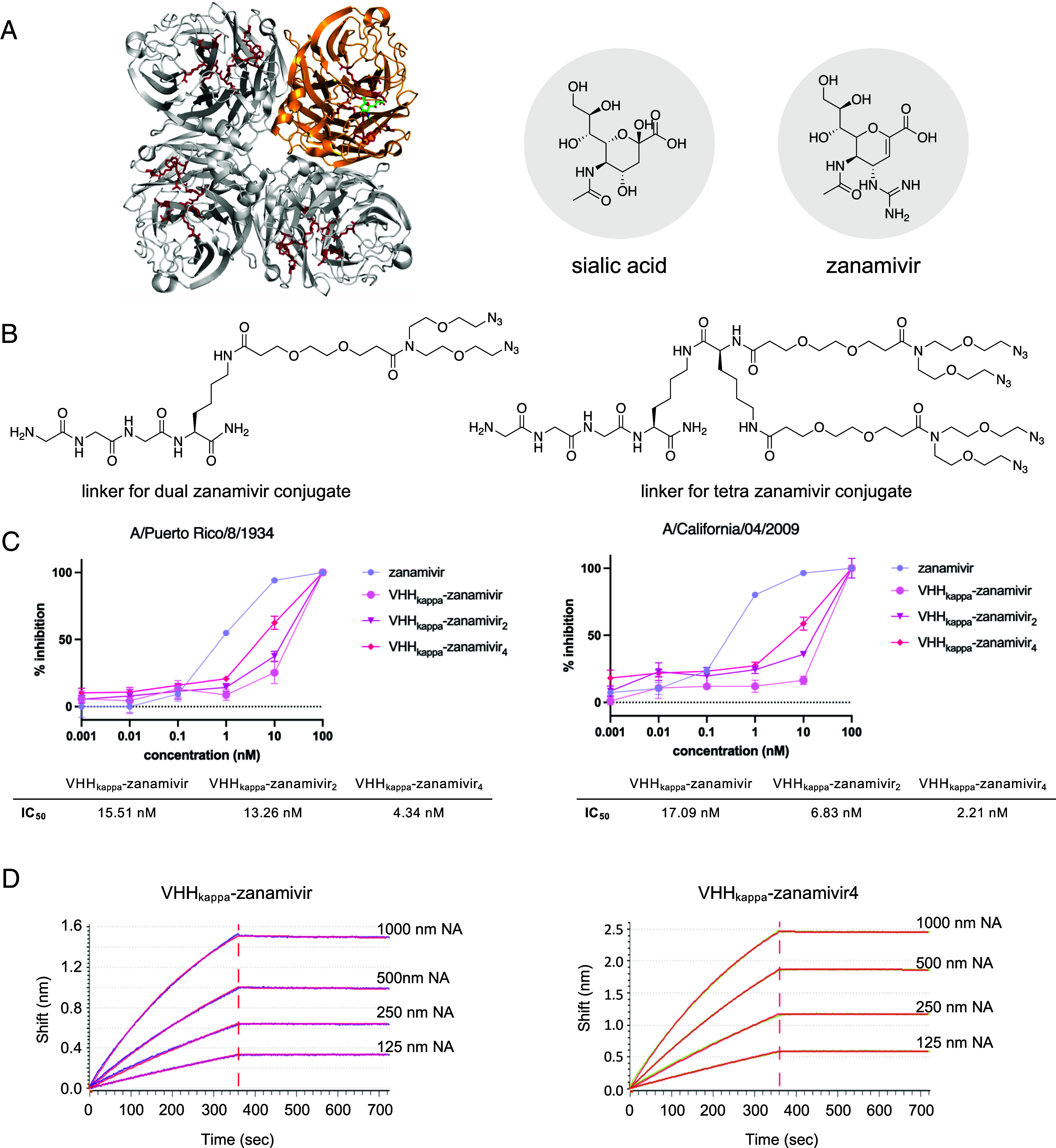
Design of VHH_kappa_–zanamivir(n) adducts and their inhibitory activity. (*A*) A ribbon diagram of the influenza virus NA (31). Residues that line the enzyme’s active site are colored red. The structure of a single NA monomer liganded by zanamivir is rendered in gold, zanamivir in green. A comparison of the structures of sialic acid and the NA inhibitor zanamivir (*Right*) underscores their structural similarity. (*B*) Design of the azide-modified sortase nucleophiles used to install two (*Left*) or four (*Right*) dibenzylcyclo-octyne modified zanamivir molecules onto VHH_kappa_. (*C*) Inhibition curves for VHH_kappa_–Zan_(n)_ and zanamivir on NA activity of influenza A/PR/8/1934 (on the *Left*) and influenza A/California/04/2009 (on the *Right*). (*D*) Real-time binding BLI. Sensorgrams of VHH_kappa_–zan and VHH_kappa_–zan4 against NA of influenza A/PR/8/1934 on GatorBio Plus BLI system.

Influenza virus NA is a homotetramer, each monomer having a binding pocket for a single zanamivir molecule (Fig. 1). The VHH_kappa_–Zan adduct previously reported carries a single molecule of drug per monomer (26). We asked whether increasing the number of zanamivir molecules per VHH_kappa_ monomer would increase the antiviral efficacy. A multivalent version of a VHH_kappa_–Zan adduct, created by increasing the number of zanamivir molecules per VHH_kappa_ monomer, would be expected to show enhanced avidity, with two possible, not mutually exclusive, outcomes: stronger inhibition of NA activity and thus a greater reduction in release of viral particles, as well as more effective recruitment of polyclonal Ig and its attendant improvement in ADCC and CDC. We hypothesize that multivalent VHH_kappa_–Zan adducts engage both mechanisms, as does their monovalent VHH_kappa_–Zan counterpart.

To obtain VHH_kappa_–Zan_2_ and VHH_kappa_–Zan_4_ (Fig. 1*B*), we applied a Sortase A-based approach for VHH bioconjugation. Through solid-phase peptide synthesis, we generated several peptide/PEG-based linkers, each modified with 1, 2, or 4 azido moieties. These flexible linkers were then sortagged onto VHH_kappa_ to generate three distinct modified VHH_kappa_-based adducts, with 1, 2, or 4 “clickable” reactive azide moieties. Following a similar synthetic route as previously described (26), zanamivir was equipped with a DBCO moiety separated by a PEG linker (Fig. 1*B*). In a copper-free click reaction, zanamivir could thus be installed on the branched nucleophiles.

The two and four zanamivir molecules are conjugated in a site-specific and flexible manner. We hypothesized that this flexible arrangement might enable simultaneous engagement of several active sites per NA tetramer. For VHH_kappa_–Zan_4_, we anticipated more complete occupancy of the active sites on NA, resulting in enhanced NA-inhibitory and antiviral activity. To assess this potential improvement, we measured NA activity in the presence of the different VHH_kappa_–Zanamivir conjugates (Fig. 1*C*). As expected, the IC_50_ of the conjugates shows a linear positive correlation with the number of zanamivir molecules conjugated to VHH_kappa_. Specifically, the IC50 of VHH_kappa_–Zan_4_ is approximately twofold lower than that of VHH_kappa_–Zan_2_ and fourfold lower than that of VHH_kappa_–Zan. It is worth noting that the VHH_kappa_-bound inhibitors exhibited reduced activity compared to “free” zanamivir, likely due to the chemical modifications at C7 employed during the conjugation process. We confirmed the enhanced binding avidity VHH_kappa_–Zan_4_ for NA by measuring the binding kinetics of VHH_kappa_–Zan and VHH_kappa_–Zan_4_ using biolayer interferometry (BLI) (Fig. 1*D*). By immobilizing the VHH conjugates on a biosensor and exposing them to varying concentrations of NA protein from the PR/8 strain, we found the dissociation constant (Kd) for VHH_kappa_–Zan to be 11 nM and for VHH_kappa_–Zan_4_ to be 3.5 nM. The multivalent VHH_kappa_–Zan_4_ conjugate thus shows significantly higher avidity for NA compared to the monovalent VHH_kappa_–Zan. The improvement in antiviral activity for VHH_kappa_–Zan_4_ exceeds its ~fourfold increase in avidity for NA, as shown below.

### VHH_kappa_–Zan_4_ Provides Complete Protection against IAV at Doses 10 Times Lower than VHH_kappa_–Zan.

Since VHHs have been delivered not only intravenously or intraperitoneally but also by inhalation (32, 33), we tested the protective effect of VHH_kappa_–Zan, VHH_kappa_–Zan_2_, and VHH_kappa_–Zan_4_ given intranasally (IN), an appealing delivery route to combat a virus that targets the respiratory tract.

**Fig. 2.**
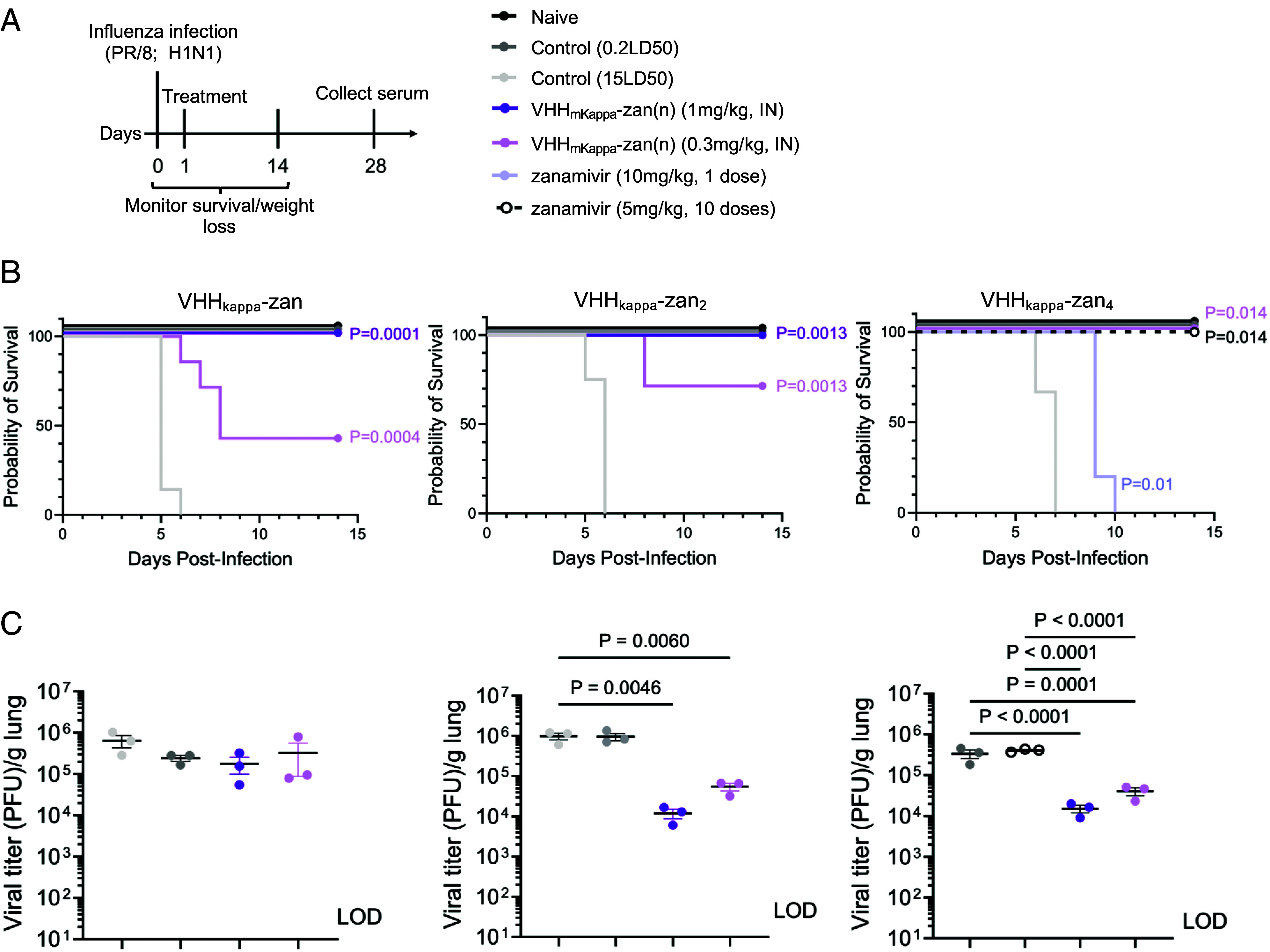
VHH_kappa_–Zan_(n)_ adducts protect against a lethal challenge with IAV. (*A*) Experimental scheme (*Top Left*) and legend for the different doses of virus, zanamivir, and the different VHH_kappa_–Zan_(n)_ adducts. (*B*) Survival curves for mice infected with PR/8 and treated with either VHH_kappa_–Zan, VHH_kappa_–Zan_2_, or VHH_kappa_–Zan_4_. (*C*) Viral titers in the lung on day 5 postinfection. Statistical significance was determined by Mantel–Cox Log rank test (*B*), or one-way ANOVA with Tukey’s multiple comparisons test (*C*). *P* values represent significance between the corresponding colored VHH_kappa_–Zan group and control (15LD50). Data represent mean ± SEM. (*B*) Survival curves (n = 5 per group). (*C*) Viral titers (n = 3 per group).

We compared the efficacy of the VHH_kappa_–Zan, VHH_kappa_–Zan_2_, and VHH_kappa_–Zan_4_ in an influenza A virus (IAV) challenge experiment. Upon infection of mice with a 15× LD50 dose of IAV, we found that VHH_kappa_–Zan_4_ was more effective than either VHH_kappa_–Zan_2_ or VHH_kappa_–Zan in preventing weight loss (*SI Appendix*) and improving survival when administered at equimolar doses 1 d after infection (Fig. 2*B*). VHH_kappa_–Zan_4_ protected 100% of mice when given at 0.3 mg/kg, while VHH_kappa_–Zan protected only 40% and VHH_kappa_–Zan_2_ protected 70% of the mice. Free zanamivir provided protection only when given at ~200 μg per day (equivalent to ~20 mg/kg) over the course of infection. Increasing the number of zanamivir molecules per VHH_kappa_ monomer thus improves protection and survival outcomes following IN administration. Intraperitoneal (IP) delivery requires higher doses to achieve the same level of protection as IN administration (*SI Appendix*). These results demonstrate that the increased inhibitory properties and avidity against NA observed for VHH_kappa_–Zan_4_ translate to increased protection from lethal IAV challenge in vivo.

Next, we measured the viral load in the lungs on day 5 postinfection. Intranasal delivery of VHH_kappa_–Zan_2_ and VHH_kappa_–Zan_4_ conjugates reduced viral titers in the lungs compared to the control group (Fig. 2*C*). Significant reductions were observed in groups treated with either 0.3 mg/kg or 1.0 mg/kg of the VHH_kappa_–Zan adduct. While free zanamivir improved survival, even twice-daily treatments failed to promote viral clearance from the lungs. This suggests that VHH_kappa_–Zan_2_ and VHH_kappa_–Zan_4_ facilitate early clearance of IAV.

**Fig. 3.**
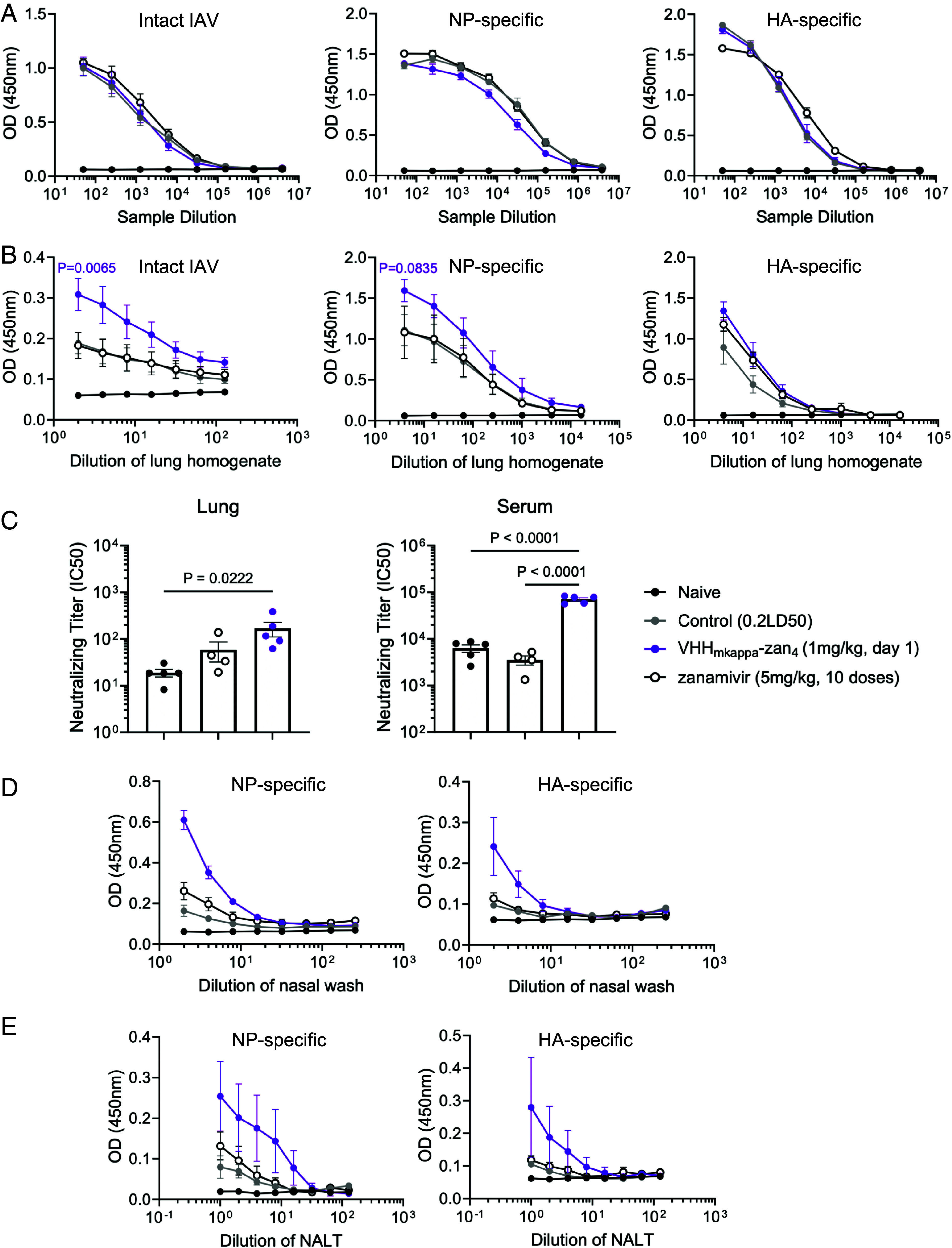
Measurement of IAV-specific and neutralizing IgG and IgA antibodies. (*A*) Serum IgG antibody titers against intact IAV PR/8 virions, PR/8 HA, and PR/8 NP. (*B*) Lung homogenate IgA antibody titers against intact IAV PR/8 virions, PR/8 HA, and PR/8 NP. (*C*) Neutralizing antibody titers against PR/8 virus in lung homogenates and serum. Neutralizing titers are expressed as the sample dilution that results in 50% inhibition of IAV plaques. (*D*) Nasal wash IgA antibody titers against PR/8 NP and PR/8 HA proteins. (*E*) NALT IgA antibody titers against PR/8 NP and PR/8 HA proteins. Statistical significance was determined by two-way ANOVA (*A* and *B*), or one-way ANOVA with Tukey’s multiple comparisons test (*C*). *P* values represent significance between the corresponding colored VHH_kappa_–Zan group and control (0.2LD50). Data represent mean ± SEM. Control (0.2LD50) (n = 5), VHH_mkappa_–Zan4 (n = 5), zanamivir (5 mg/kg, 10 doses) (n = 4), naive (n = 3).

The mechanism of action of VHH_kappa_–Zan(n) conjugates involves engagement of the kappa light chain of a highly diverse repertoire of immunoglobulins and may improve engagement of the immune system by ADCC and/or CDC. This might lead to higher titers of IAV-specific antibodies in the treated mice and to what is referred to as a “vaccinal effect.” To test this hypothesis, we measured antibody titers against intact IAV and two immunogenic IAV proteins, the hemagglutinin (HA) and nucleoprotein (NP), in mice treated with 1 mg/kg of VHH_kappa_–Zan_4_. Interestingly, no significant differences in IAV-specific serum IgG titers were observed between VHH_kappa_–Zan_4_ treated mice and mice that received twice-daily doses of zanamivir or the control group that received a sublethal IAV infection (Fig. 3*A*). As the adduct is administered intranasally, it is possible that any enhancement in IAV-specific immunity occurs at the site of administration. Thus, we assessed the IgA antibody titers within the lung tissue. IgA plays a crucial role in mucosal immunity and provides a critical first response against respiratory pathogens. We observed a significant increase in IgA titers in the lung homogenates of mice treated with VHH_kappa_–Zan_4_ compared to controls, including animals treated with free zanamivir (Fig. 3*B*). Next, when we assessed the IAV-neutralizing capacity of antibodies in mice treated with VHH_kappa_–Zan_4_, both systemic IgG and lung-localized IgA showed improved neutralizing activity against live IAV compared to the controls (Fig. 3*C*). Finally, we evaluated the IgA antibody titers in the upper respiratory tract, in both nasal washes collected by flushing saline through the nasal passages and in the nasal-associated lymphoid tissue (NALT). The titers of IgA were higher in mice treated with VHH_kappa_–Zan_4_ compared to control groups, suggesting that IN administration of VHH_kappa_–Zan_4_ promotes enhanced antibody responses in the upper respiratory mucosa (Fig. 3 *D* and *E*). Early administration of VHH_kappa_–Zan_4_ thus provides a vaccinal effect by enhancing the generation of the IAV-specific humoral immune response.

### VHH_kappa_–Zan_4_ Does Not Enhance the IAV-Specific T Cell Response.

An influenza virus-specific T cell response precedes the development of the virus-specific antibody response and plays a role in viral clearance (34). To test whether there is a T cell-mediated vaccinal effect of the VHH_kappa_–Zan_4_, we infected mice with a lethal dose of IAV and treated them with VHH_kappa_–Zan_4_ 1 d later. Seven days postinfection, we harvested the lungs and analyzed the presence of IAV-specific T cells. Using the following influenza-specific tetramers: H-2D^b^-restricted NP_366-374_, H-2D^b^ restricted Polymerase Acidic Protein PA_224-233_, and I-A^b^-restricted NP_311-325_, we enumerated the IAV-specific CD4+ and CD8+ T cell populations. We saw no significant differences in these populations when comparing VHH_kappa_–Zan_4_ treated mice with those that survived owing to treatment with free zanamivir or with mice infected with a sublethal dose of IAV (*SI Appendix*).

### VHH_kappa_–Zan_4_ Treatment Provides Complete Protection against IAV 3 Days Post Infection.

**Fig. 4.**
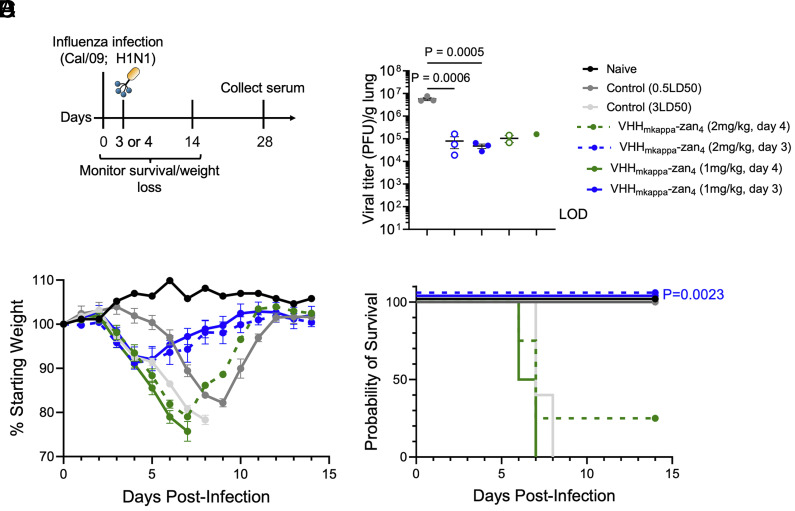
Late administration of VHH_kappa_–Zan_4_ protects against a lethal IAV challenge. (*A*) Experimental scheme. (*B*) Viral titers in the lung on day 5 postinfection. (*C* and *D*) Weight loss and survival curves for mice infected with Cal/09 and treated with VHH_kappa_–Zan_4_ on day 3 or 4 postinfection. Statistical significance was determined by one-way ANOVA with Tukey’s multiple comparisons test (*B*), or Mantel–Cox Log rank test (*D*). Data represent mean ± SEM. Each experimental condition starts with n = 5 per group. Only a subset of mice is available for further analysis due to mortality within the groups. Out of 5 mice, no mice survived in the VHH_mkappa_–Zan4 (1 mg/kg, day 4) group, only 2 mice survived in the VHH_mkappa_–Zan4 (2 mg/kg, day 4) group, all 5 survived in the VHH_mkappa_–Zan4 (1 mg/kg, day 3) group, all 5 survived in the VHH_mkappa_–Zan4 (1 mg/kg, day 3) group, and all 5 survived in the control (0.2LD50) group.

Conventional antiviral treatments including zanamivir demonstrate the highest efficacy when given early after infection. We examined how far we could delay treatment and still maintain protection against a lethal IAV challenge. We found that VHH_kappa_–Zan_4_ given on either day 3 or 4 of infection with IAV promoted early viral clearance in the lungs (Fig. 4*B*). This is all the more striking given that there was only a 24-h window between the treatment on day 4 and the assessment of viral titers on day 5. Treatment with either 2 mg/kg or 1 mg/kg VHH_kappa_–Zan_4_ on day 3 rescued mice from further weight loss and protected 100% of mice from IAV-induced death (Fig. 4 *C* and *D*). Finally, a 2 mg/kg dose of VHH_kappa_–Zan_4_ given on day 4 postinfection protected 25% of mice from IAV-induced death. Thus, treatment can be delayed until day 3 postinfection while maintaining a robust efficacy against a lethal IAV challenge.

### Prophylactic Administration of VHH_kappa_–Zan_4_ Provides Extended Protection against a Lethal IAV Challenge.

**Fig. 5.**
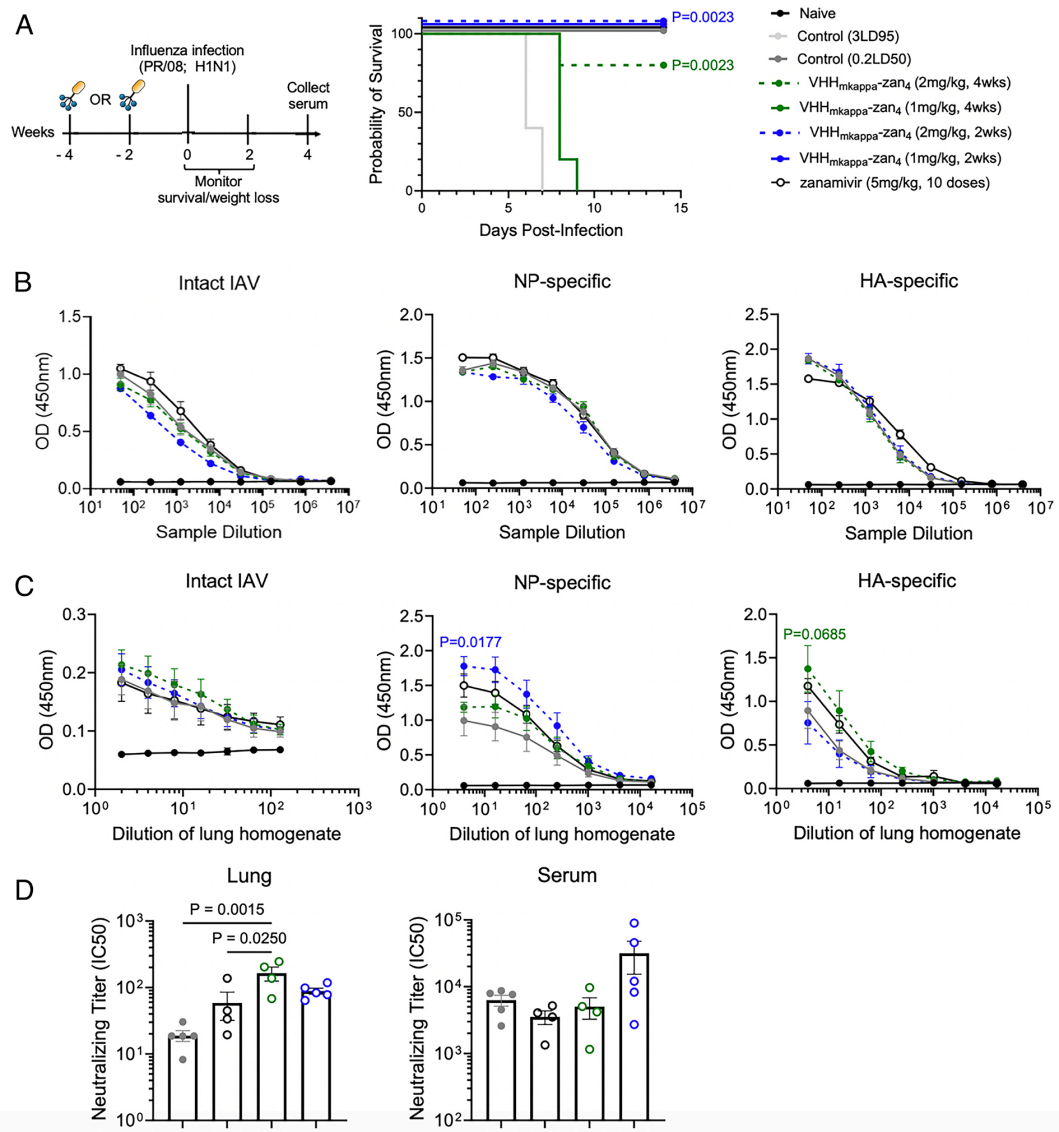
Prophylactic administration of VHH_kappa_–Zan_4_ provides extended protection against IAV. (*A*) Experimental scheme and legend to the different experimental conditions (*Left*). Survival curves for VHH_kappa_–Zan_4_ treated mice (*Left*). (*B*) Serum IgG antibody titers against intact IAV PR/8 virions, PR/8 NP, and PR/8 HA. (*C*) Lung homogenate IgA antibody titers against intact IAV PR/8 virions, PR/8 NP, and PR/8 HA. (*D*) Neutralizing antibody titers against PR/8 virus in lung homogenates and serum. Neutralizing titers are expressed as the sample dilution that results in 50% inhibition of IAV plaques (IC50). Statistical significance was determined by Mantel-Cox Log rank test (*A*), two-way ANOVA (*B* and *C*), or one-way ANOVA with Tukey’s multiple comparisons test (*D*). Data represent mean ± SEM. Each experimental condition starts with n = 5 per group. Only a subset of mice is available for further analysis due to mortality within the groups. Out of 5 mice, no mice survived in the VHH_mkappa_–Zan4 (1 mg/kg, 4wk) group, four mice survived in the VHH_mkappa_–Zan4 (2 mg/kg, 4wk) group, all 5 survived in the VHH_mkappa_–Zan4 (1 mg/kg, 2wk) group, all 5 survived in the VHH_mkappa_–Zan4 (1 mg/kg, 2wk) group, and all 5 survived in the control (0.2LD50) group.

The circulatory half-life of immunoglobulins is a key determinant of how long protection afforded by VHH_kappa_–Zan_(n)_ conjugates lasts (26). The VHH_kappa_–Zan adduct given intraperitoneally protects mice from a lethal dose of IAV when given up to 7 d prior to infection (26). We performed experiments to determine the allowable delay between intranasal (IN) delivery of VHH_kappa_–Zan_4_ and an IAV challenge. Animals received a single IN dose of VHH_kappa_–Zan_4_ at 1 or 2 mg/kg, either 4 or 2 wk prior to a challenge with 15xLD50 of IAV. Mice that received VHH_kappa_–Zan_4_ at 2 wk before infection, regardless of the dose, afforded full protection (Fig. 5*A*). While mice that received a single 1 mg/kg dose 4 wk before infection did not survive the challenge, 80% of those that received 2 mg/kg VHH_kappa_–Zan_4_ survived. Similarly, to mice that were treated 24 h postinfection, no improvement was observed in serum IgG antibody titers of those treated 2 or 4 wk prior to infection (Fig. 5*B*). Animals treated with VHH_kappa_–Zan_4_ 2 wk previously did show increased NP-specific IgA in lung samples, as well as increased neutralizing activity in both serum and lung samples (Fig. 5 *C* and *D*). The average half-life of mouse immunoglobulins is only ~7 d, much shorter than the ~30-d half-life typical in humans. These results suggest the potential for the development of an adduct that could protect human patients for a period of months.

**Fig. 6.**
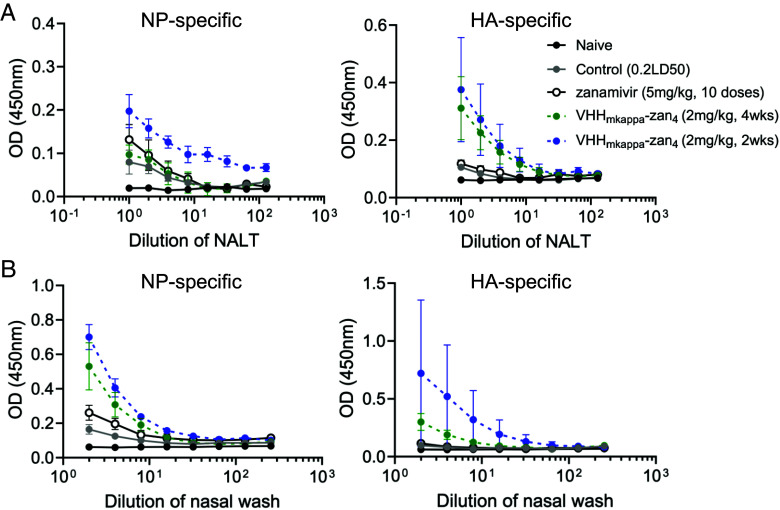
Prophylactic administration of VHH_kappa_–Zan_4_ promotes enhanced mucosal IgA antibody titers. (*A*) Nasal wash IgA antibody titers against PR/8 NP and PR/8 HA proteins. (*B*) NALT IgA antibody titers against PR/8 NP and PR/8 HA proteins. Data represent mean ± SEM. Control (0.2LD50) (n = 5), VHH_mkappa_–Zan4 (2 mg/kg, 4wk) (n = 4), VHH_mkappa_–Zan4 (2 mg/kg, 2wk) (n = 5), zanamivir (5 mg/kg, 10doses) (n = 4), naive (n = 3).

Finally, we evaluated the IgA antibody titers in the upper respiratory tract in mice that were prophylactically treated with 2 mg/kg of VHH_kappa_–Zan_4_ 2 or 4 wk prior to an IAV infection. Mice that received VHH_kappa_–Zan_4_ at 2 wk before infection demonstrated enhanced IgA titers specific for the NP and HA proteins in both the NALT and in fluid collected from the nasal passages (Fig. 6 *A* and *B*). Animals treated 4 wk prior to infection showed more modestly increased IgA titers compared to controls. These data suggest that IN administration of VHH_kappa_–Zan_4_ promotes enhanced antibody responses in the upper respiratory tract. Overall, these data demonstrate that intranasal treatment with VHHkappa–Zan4 can be utilized both prophylactically or therapeutically to provide complete protection against a lethal IAV infection.

### Concluding Remarks.

The approach described here for the various modifications of VHH_kappa_ merits further comment. First, no immunization is required for VHH_kappa_–Zan_(n)_ to reap the benefits of all effector functions associated with the various Ig isotypes and subclasses, regardless of antibody specificity. Second, the small molecule zanamivir, used here as a recognition module, can be swapped out for other entities, including nanobodies (26). Any small molecule (or nanobody/antibody fragment) capable of interacting with the surface of a pathogen or pathogen-infected cell deserves exploration as a modifier of VHH_kappa_ to achieve recruitment of Ig. However, increasing the size of these types of adduct, as would be the case upon installation of multiple nanobodies, may compromise the possibility of intranasal delivery. Third, the recognition unit, be it a small molecule or a nanobody, need not be neutralizing or otherwise have antiviral or antibacterial properties on its own, as long as it enables the recruitment of immunoglobulins to the pathogen or infected cell. Fourth, the efficacy of these VHH_kappa_ adducts, to a first approximation, lasts as long as their complex with circulating immunoglobulins persists (26). This means that in mice, where the circulatory half-life of immunoglobulins is several days to a week, a dose can be chosen that retains activity for several half-lives, thus extending protection to at least weeks. If transposed to the human setting -and using VHHs that recognize human kappa light chains- protection is expected to last much longer, based on the circulatory half-life of human Ig, which is measured in weeks (35, 36). VHH_kappa_–Zan_(n)_ adducts are covalent conjugates via stable amide and carbamate linkages, without relying on maleimide-type linkers. They are expected to survive as long as the adduct is present, although this remains to be verified by experiment. Fifth, protection against the targeted pathogen is achieved very soon after delivery of the administered modified VHH_kappa_. Both prophylactic and therapeutic applications are therefore possible, as no induction of an immune response is required. Sixth, the modest size of VHH_kappa_–Zan_(n)_ is most likely responsible for its ability to exert its effect when administered intranasally. We have not examined whether intranasal delivery of VHH_kappa_ fused to another VHH would still be compatible with protection. In conclusion, it is clear that by increasing the number of zanamivir molecules per VHH_kappa_, we have produced an antiviral that can be given intranasally, the effects of which last much longer than that of the small molecule delivered on its own.

Finally, in preliminary experiments, we have shown that this approach is not limited to the elimination of virus and virus-infected cells. Fusions of VHH_kappa_ with anti-Class II MHC VHHs efficiently reduce the number of Class II MHC+ cells in vivo, while fusions of VHH_kappa_ with an anti CTLA-4 VHH result in starkly reduced tumor growth through a reduction in the number of intra tumoral regulatory T cells (37).

## Materials and Methods

### Influenza Viruses.

Influenza virus A/Puerto Rico/8/1934 (H1N1) (NR-348) and A/California/07/2009 (H1N1) (NR-13663) were obtained from BEI resources and propagated in MDCK cells (American Type Culture Collection [ATCC], Manassas, VA).

### NA Inhibition Assay.

Inhibition of influenza NA activity was assayed using the NA-Fluor Influenza NA Assay Kit (Thermo Fisher). Briefly, influenza H1N1 viruses A/Puerto Rico/8/1934 and A/California/07/2009 were assayed at serial twofold dilutions of 1:1-1:1,024 to determine an appropriate working concentration in the linear portion of the NA activity curve. Using these viruses at a 1:4 dilution (after dilution: 6.5 × 10^5^ PFU/mL of A/Puerto Rico/8/1934 and 1.5 × 10^3^ PFU/mL of A/California/07/2009), the various VHH–Zanamivir conjugates were tested against the respective concentration of zanamivir to assess NA activity inhibition. For each drug, a 50% NA inhibitory concentration (IC_50_) value was calculated by fitting a sigmoidal dose–response (four-point logistic) curve using GraphPad Prism 10 software.

### Mouse Experiments.

8 to 10-wk-old female C57Bl/6 mice were purchased from the Jackson Laboratory and housed in the animal facility at Mispro Biotech Services. All experimental procedures using mice were approved and carried out in accordance with Institutional Animal Care and Use Committee (IACUC) under protocol #2023-CRB-03. Euthanasia was performed according to the guidelines of IACUC and were consistent with the American Veterinary Medicine Association (AVMA) Guidelines on Euthanasia. Mice were infected IN with either A/Puerto Rico/8/1934 (PR/8) or A/California/07/2009 (Cal/09) virus while anesthetized with isoflurane. Infected mice were monitored daily for weight loss and mortality and humanely euthanized at a threshold of 80% weight loss. When indicated, mice received one intranasal dose of VHH_kappa_ adduct at the indicated concentration in 75 μL volume while anesthetized with isoflurane. Intraperitoneal administration of the VHH_kappa_ adduct was given in 100 μL total volume diluted in sterile PBS. Zanamivir-treated groups received either one IN dose at 10 mg/kg administered 24 h postinfection or received a 5 mg/kg IN dose twice per day during the first 5 d of infection.

### Plaque Assay for Viral Titers.

Whole lungs were homogenized, and supernatant was flash frozen and stored at −80 °C until further analysis. Samples were diluted fourfold starting at 1:10 then added to MDCK cells in six-well plates and incubated at 37 °C for 1 h. Wells were washed with PBS and overlaid with 1:1 mixture 2× Eagle MEM (Quality Biological) and 1.6% SeaKem ME agarose (Lonza) supplemented with 2 μg/mL TPCK-trypsin. Plates were incubated for 3 d at 37 °C with 5% CO_2_. Following incubation, the agar plug was removed from wells and cells were fixed with 70% Ethanol. Cells were stained with a 1% crystal violet solution and viral plaques were counted after 24 h.

### Antibody ELISA.

Serum, lung homogenates, and NALT were collected on day 28 postinfection and stored at −80 °C until further analysis. Nasal wash fluid was collected by cannulation of the trachea, and 300 μL of sterile PBS was washed through the nasal cavity, collected out the nose, and stored at −80 °C. Polystyrene high-binding plates (Corning) were coated overnight at 4 °C with either live A/Puerto Rico/8/34 virus (1 × 10^4^ PFU/well), A/Puerto Rico/8/34 NP (1 μg/mL) (Sino Biological), or A/Puerto Rico/8/34 HA (1 μg/mL) (Sino Biological). Plates were blocked with 5% nonfat dry milk in PBS for 1 h at 37 °C. Samples were serially diluted fivefold starting at 1:50 (serum) or 1:4 (lung), and plates were incubated overnight at 4 °C. Goat anti-mouse HRP-conjugated antibody specific for IgG or IgA (Southern Biotech) was added at 1:4,000 dilution and incubated for 1 h at 37 °C. Plates were developed in 3,3′,5,5′-tetramethylbenzidine solution (Sigma-Aldrich), and the reaction was stopped with ELISA stop solution (Thermo Fisher). Absorbance values were measured at 450 nm.

### Neutralizing Antibody Titers.

Serum or lung homogenates were collected on day 28 postinfection and stored at −80 °C until further analysis. Samples were diluted fivefold starting at 1:50 (serum) or 1:4 (lung) and mixed with 500 PFU IAV PR/8 in a 96-well round bottom plate prior to incubation at 37 °C for 1 h. 100 μL of virus/sample mixture was added to confluent MDCK cells in 6-well plates and further incubated at 37 °C for 1 h. Plates were washed, overlaid, and stained as described above for the viral titer plaque assay. A four-parameter fit curve analysis was used to determine the serum dilution that resulted in 50% inhibition of IAV viral plaques.

### Antibody Staining and Flow Cytometry.

Whole lungs were digested in 4 mL RPMI supplemented with 0.5 mg/mL DNase I (Sigma-Aldrich) and 2.5 mg/mL collagenase I (Sigma-Aldrich) for 30 min at 37 °C. Digested lungs were homogenized with an AutoMACS (Miltenyi) and passed through a 70 μm filter to generate a single-cell suspension. Cells were stained with live/dead Fixable Aqua (Thermo Fisher) for 30 min at 4 °C. For tetramer staining, cells were stained with either IAV-specific NP_311-325_ (4 μg/mL) for 3 h at 37 °C, or IAV-specific NP_366-374_ (2 μg/mL) and IAV-specific PA_224-233_ (2 μg/mL) for 30 min at 4 °C. Extracellular staining was then performed using antibodies for CD45.2 (104; Thermo Fisher), CD90.2 (53-2.1; Thermo Fisher), CD11a (M17/4; Thermo Fisher), CD44 (IM7; Thermo Fisher), CD4 (RM4-5; Thermo Fisher), and CD8 (53-6.7; Thermo Fisher) for 30 min at 4 °C. For intracellular cytokine staining, cells were stimulated with eBioscience cell stimulation cocktail (Thermo Fisher) for 5 h followed by staining for live/dead Fixable Aqua and extracellular markers as described above. Cells were fixed using eBioscience Intracellular Fixation and Permeabilization Buffet set (Thermo Fisher) followed by staining with antibodies for IFN-γ (XMG1.2; Thermo Fisher) and TNFα (MP6-XT22; Thermo Fisher) for 30 min at 4 °C. Samples were run on an Attune CytPix (Thermo Fisher) and analyzed using FlowJo software (BD Biosciences).

### Synthesis Mono, Dual, and Tetra Azido-Linkers.

Peptide-based linkers were synthesized manually using standard Fmoc–SPPS chemistry protocol. The following protected-amino acids with side-chain protection groups were used: Fmoc–Lys(N3)–OH, Fmoc–Lys(mtt)–OH, Boc–Gly–Gly–Gly–OH, Fmoc–Lys(Fmoc)–OH as well as the azide-carrying PEG linker: *N*,*N*-Bis(PEG1-azide)–*N*-PEG2–acid. SPPS was performed on Rink-amide polystyrene resin. Manual loading of the first amino acid residue on the resin and subsequent Fmoc–SPPS, followed established standard protocols.

In summary, Fmoc-deprotections were performed with 20% piperidine in DMF. 2 × 8 min. Couplings were performed with Fmoc–amino acid (4.0 equiv relative to resin substitution), HATU (3.9 equiv), and DIPEA (8.0 equiv) in DMF for 45 min. For coupling of the azido-PEG linker, the reaction time was extended to 90 min.

The completed peptide was cleaved from the Rink Amide polystyrene resin using a cleavage cocktail of 95:2.5:2.5 TFA:TiPS:H2O and shaken for 2 h. The resin was removed by filtration and washed with TFA (5 mL/g resin), the filtrate was placed in a plastic centrifugal tube (40 mL), and volatiles were removed under reduced pressure. The residue was triturated with Et2O (ca. 30 mL/g resin), centrifuged (3,500 g, 3 min), and the supernatant was removed by decantation. The crude material was dried using N2 flow and dissolved in a suitable solvent (1:1 CH3CN:H2O + 0.1% TFA) for RP-HPLC purification.

The linkers were purified by reverse phase high-performance liquid chromatography (RP-HPLC) The mobile phase for RP-HPLC were Milipore-H2O containing 0.1% TFA and HPLC-grade CH3CN containing 0.1% TFA. Preparative HPLC was performed on a C18 column (5 μm, 100 Å pore size, 20 mm I.D. × 250 mm), at the flow rate of 20 mL/min.

The purified fractions containing the desired product were combined and lyophilized to yield the desired product as a white powder.

### Synthesis of Zanamivir-PEG_6_-DBCO.

Zanamivir-PEG_6_-DBCO was prepared following the previously described Procedure (ref). To a THF solution containing the protected and carbonyldiimidazole-activated zanamivir compound, DBCO-PEG6-amine and diisopropylethylamine were added and stirred overnight at room temperature. The final step is identical to the previously described work (ref). LC-MS [M + H]+ = 970.04.

### VHH_kappa_ Production.

VHH_kappa_ was cloned into a pHEN6 and recombinantly expressed in WK6 *Escherichia coli*. Cells were then grown at 37 °C in Terrific Broth containing ampicillin (100 mg/L) until the optical density at 600 nm (OD600) reached 0.6 to 0.8. To induce the VHH expression, 1 mM isopropyl-β-D-thiogalactopyranoside was added, and incubation was continued overnight at 30 °C. Cells were harvested by centrifugation. VHHs were released by osmotic shock using tris/EDTA/ sucrose (TES) buffer [200 mM tris, 0.65 mM EDTA, and 0.5 M sucrose (pH 8)].

VHHs were isolated on Ni-NTA beads and further purified by size exclusion chromatography using a Superdex 75 10/600 column. To deplete lipopolysaccharide (LPS), purified VHHs were reloaded on Ni-NTA beads, which were then washed with PBS solution (40 column volumes) containing 0.1% (v/v) Triton X-114. Elution was performed with endotoxin-free PBS containing 500 mM imidazole. Imidazole was removed by desalting on a PD10 column using LPS-free PBS as the elution buffer.

### VHH_kappa_–Zanamivir.

VHH_kappa_-LPETGGHHHHHH was first modified with the desired linker (mono, dual, and tetra azido-linker) using sortase A mediated ligation. The general procedure is as follows: to 0.5 mL of VHHkappa-LPETGGHHHHHH in PBS (250 μM) was added the 50 μL of the desired linker (1.0 mM) and 40 μL of sortase A 7 M (0.5 mM). The resulting mixture was stirred overnight at 15 °C. The completion of the reaction was monitored by LCMS and when completion reached over 80%, the reaction mixture was filtered through Ni-NTA resin to remove the sortase, the unreacted VHHkappa, and the cleaved histidine tag. The remaining excess of the linker was removed via PD10 column to afford the clean VHHkappa-linker-(N_3_)n.

The final conjugation was done by mixing into a PBS solution of VHHkappa-linker-(N_3_)n (100 μM), 1.5 equivalent of zanamivir-PEG_6_-DBCO compared to n. The reaction was stirred at 15 °C, and the completion was monitored by LCMS. The remaining excess of zanamivir-PEG_6_-DBCO was removed by PD10 column or by size exclusion chromatography depending on the scale of the reaction.

For example, to prepare VHHkappa–Zan4, 200 μL of VHHkappa-linker-(N_3_)_4_ (100 μM) in PBS was mixed with 40 μL of DBCO-PEG–Zanamivir (2.4 mM) and stirred at 15 °C. Completion was reached after 20 h, and the reaction mixture was passed through PD10 column to remove the excess of DBCO–PEG–zanamivir.

## Supplementary Material

Appendix 01 (PDF)

## Data Availability

All study data are included in the article and/or *SI Appendix*.
