# Supplementary material for: A multivalent nanobody–drug conjugate to prevent and treat influenza virus infections
Source: Proc Natl Acad Sci U S A. 2025 Nov 3;122(45):e2409565122. doi: 10.1073/pnas.2409565122 (PMC12625891; doi:10.1073/pnas.2409565122)
Supplement: Supplementary file 1 — Appendix 01 (PDF) [file pnas.2409565122.sapp.pdf]

PNAS

Supporting Information for

A Multivalent Nanobody-Drug Conjugate to Prevent and Treat Influenza Virus Infections.

Thibault J. Harmand[1*], Laura Pietrok[1*], Helen Rich[1], Rhogerry Deshycka[1], Laney Flannagan[1], Aaron Accardo[1], Novalia Pishesha[2,3,#], Hidde L. Ploegh[4,#]

[1]Cerberus Therapeutics, Cambridge, MA, USA
[2]Division of Immunology, Boston Children's Hospital, Boston, MA, USA
[3]Department of Pediatrics, Harvard Medical School, Boston, MA, USA
[4]Program in Cellular and Molecular Medicine, Boston Children's Hospital, Boston, MA, USA
[*] T.J.H. and. L.P. contributed equally to this work.

To whom correspondence may be addressed. E-mail: hidde.ploegh@childrens.harvard.edu or novalia.pishesha@childrens.harvard.edu

This PDF file includes:

Figures S1 to S6

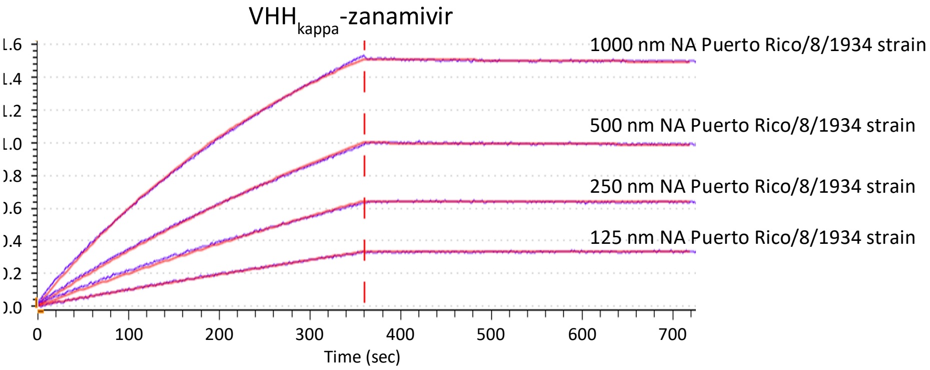

Experiment	NA Puerto Rico/8/1934 M Conc (nM)	KD(M)	Rmax	Req	Loading Height	FullR2	Full X2
VHHkappa-zanamivir1 Against NA Puerto Rico/8/1934	1000	1.08E-008	2.3043	2.2798	0.6055	1	0.921
	500	1.08E-008	2.4134	2.3625	0.5909	1	0.921
	250	1.08E-008	2.7405	2.6275	0.5863	1	0.921
	125	1.08E-008	2.6545	2.4441	0.6096	1	0.921

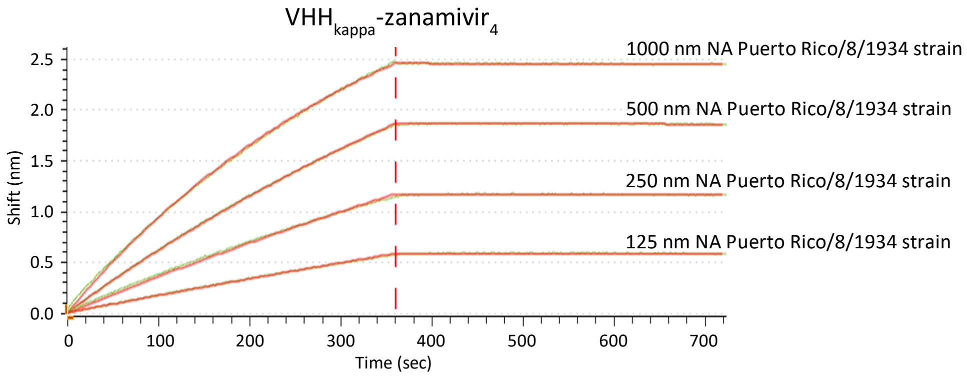

Experiment	NA Puerto Rico/8/1934 M Conc (nM)	KD(M)	Rmax	Req	Loading Height	FullR2	Full X2
VHHkappa-zanamivir1 Against NA Puerto Rico/8/1934	1000	3.56E-009	3.9408	3.9268	0.611	1	1.454
	500	3.56E-009	4.824	4.7898	0.6046	1	1.454
	250	3.56E-009	5.396	5.3202	0.5978	1	1.454
	125	3.56E-009	5.0731	4.9325	0.6007	1	1.454

Fig. S1. Real-time binding Biolayer interferometry (BLI). Sensorgrams of VHH_{kappa}-zan and VHH_{kappa}-zan4 against NA of influenza A/PR/8/1934 on GatorBio Plus BLI system. The VHH-Zanamivir conjugates were immobilized onto anti-VHH sensors and immersed in NA -containing buffer at different concentrations. VHH-Zanamivir K_D (nM) = 11. VHH-Zanamivir-4 K_D (nM) = 3.5.

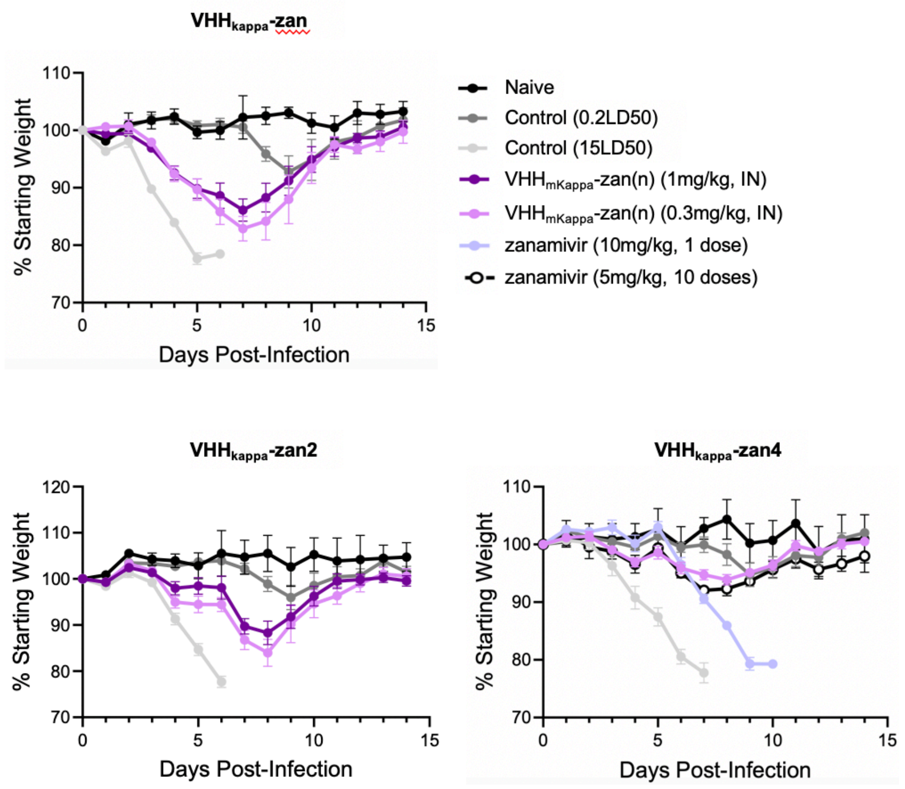

Fig. S2. Weight loss curves of mice treated with either a single dose at 1mg/kg or 0.3mg/kg of VHHkappa-zan, VHHkappa-zan2 or VHHkappa-zan4.

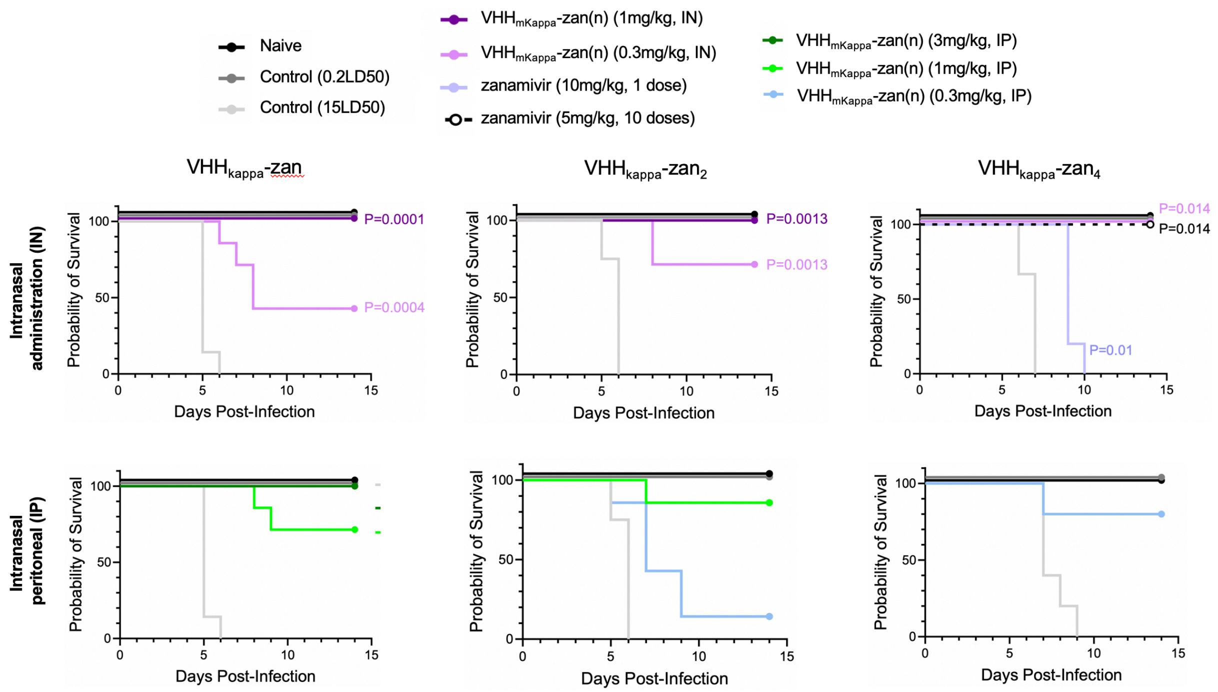

Fig. S3. Survival curves of mice treated with varying doses of three different conjugates. Comparison of survival outcomes between two routes of administration: intraperitoneal (IP) and intranasal (IN).

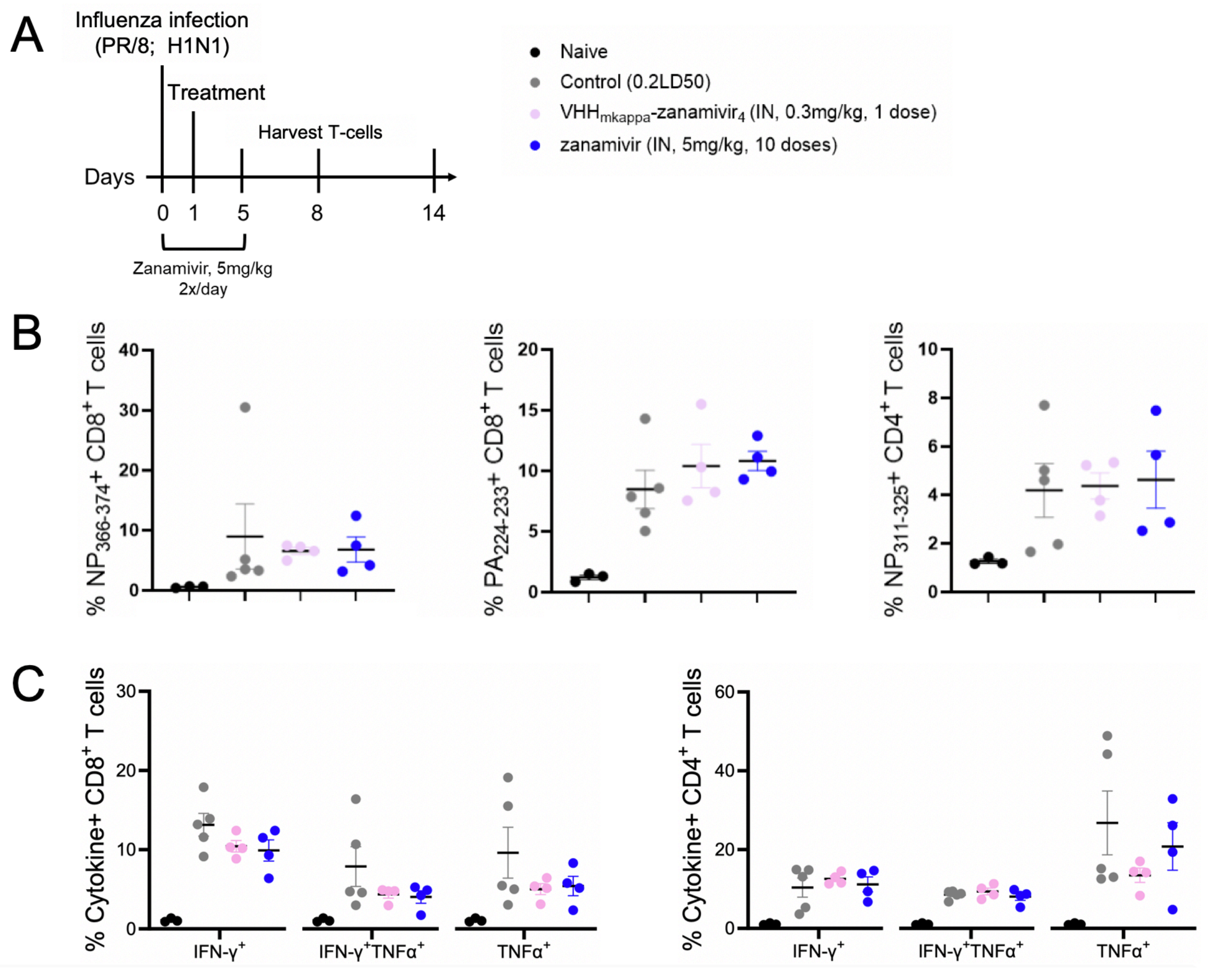

Fig. S4. Characterization of the IAV-specific T cell response and cytokine production in animals infected with IAV and treated with VHH_{kappa}-Zan4 adducts. A: Experimental scheme and legend to the various conditions. **B:** The frequencies of tetramer-positive CD8 T cells and CD4 T cells in the lungs as measured by flow cytometry. **C:** The relative numbers of CD8 and CD4 T cells that produce IFNγ, TNFα or both).

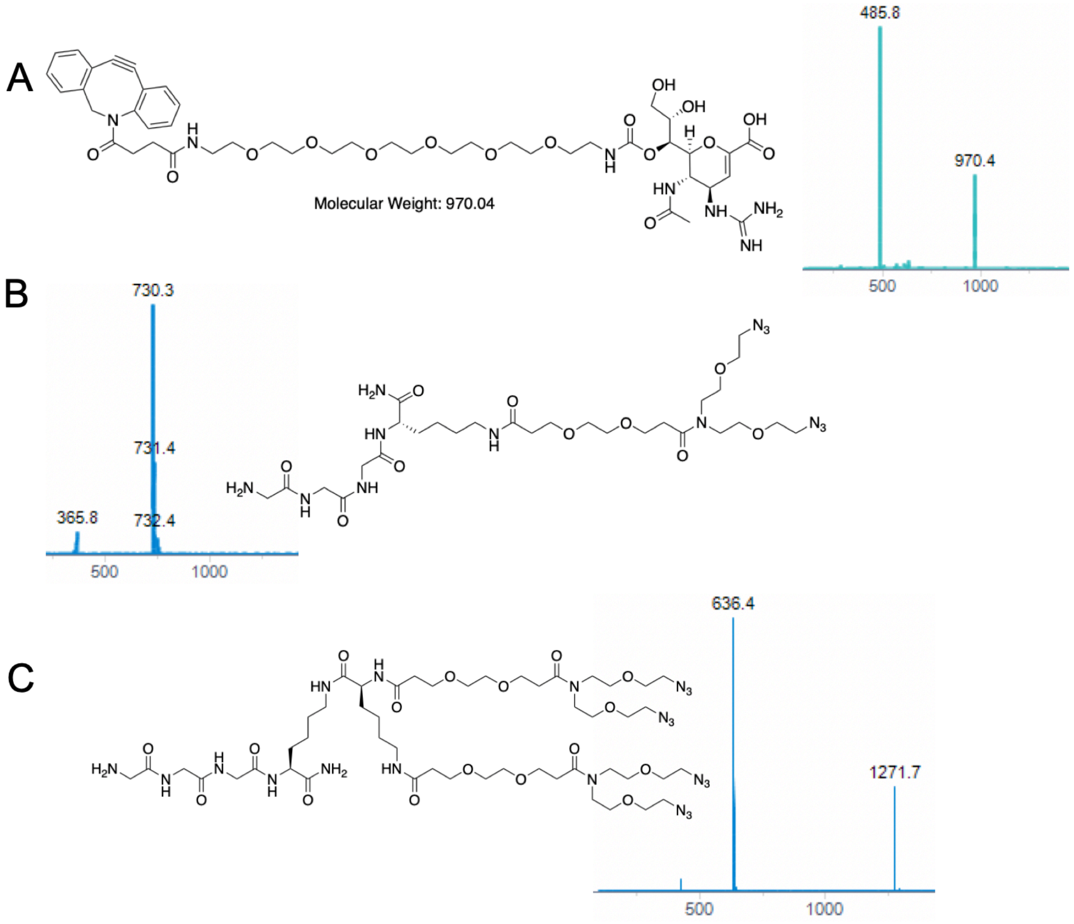

Fig. S5. Chemical structure and LCMS traces of **A**: zanamivir-DBCO (Calc Mw: 970, found Mw: 970 (M), 485 (M/2)), and sortase-ready **B**: dual (Calc Mw: 729, found Mw: 730 (M), 365 (M/2)) and **C**: tetravalent linkers (Calc Mw: 1271, found Mw: 1271 (M), 636 (M/2))

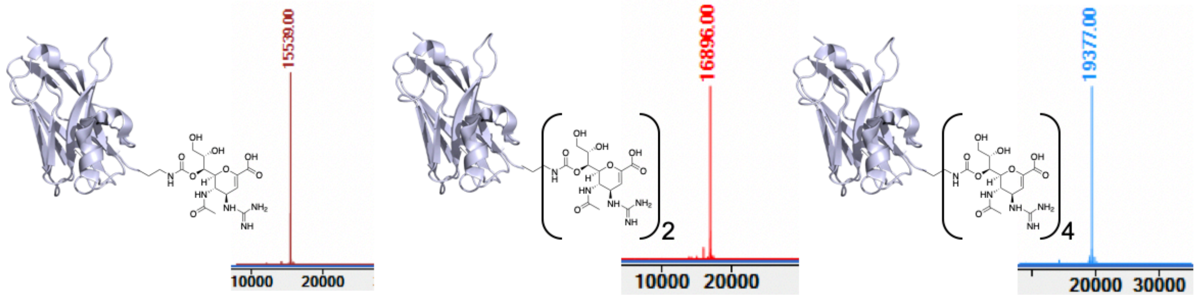

VHH$_{kappa}$-zan **VHH$_{kappa}$-zan$_2$** **VHH$_{kappa}$-zan$_4$**

Fig. S6. LCMS traces of VHH$_{mousekappa}$-zanamivir (Calc Mw: 15540, found Mw: 15539), VHH$_{kappa}$-zanamivir$_2$ (Calc Mw: 16898, found Mw: 16896) and VHH$_{kappa}$-zanamivir$_4$ (Calc Mw: 19378, found Mw: 19377).
